# Optics of cone photoreceptors in the chicken (*Gallus gallus domesticus*)

**DOI:** 10.1098/rsif.2015.0591

**Published:** 2015-10-06

**Authors:** David Wilby, Matthew B. Toomey, Peter Olsson, Rikard Frederiksen, M. Carter Cornwall, Ruth Oulton, Almut Kelber, Joseph C. Corbo, Nicholas W. Roberts

**Affiliations:** 1 School of Biological Sciences, University of Bristol, Bristol Life Sciences Building, Tyndall Avenue, Bristol BS8 1TQ, UK; 2 H.H. Wills Physics Laboratory, University of Bristol, Tyndall Avenue, Bristol BS8 1TL, UK; 3 Bristol Centre for Functional Nanomaterials, Centre for Nanoscience and Quantum Information, University of Bristol, Tyndall Avenue, Bristol BS8 1FD, UK; 4 Department of Pathology and Immunology, Washington University School of Medicine, St Louis, MO, USA; 5 Vision Group, Department of Biology, Lund University, Sölvegatan 35, Lund, Sweden; 6 Department of Physiology and Biophysics, Boston University School of Medicine, Boston, MA, USA

**Keywords:** cone, photoreceptors, birds, colour vision, optics, FDTD

## Abstract

Vision is the primary sensory modality of birds, and its importance is evident in the sophistication of their visual systems. Coloured oil droplets in the cone photoreceptors represent an adaptation in the avian retina, acting as long-pass colour filters. However, we currently lack understanding of how the optical properties and morphology of component structures (e.g. oil droplet, mitochondrial ellipsoid and outer segment) of the cone photoreceptor influence the transmission of light into the outer segment and the ultimate effect they have on receptor sensitivity. In this study, we use data from microspectrophotometry, digital holographic microscopy and electron microscopy to inform electromagnetic models of avian cone photoreceptors to quantitatively investigate the integrated optical function of the cell. We find that pigmented oil droplets primarily function as spectral filters, not light collection devices, although the mitochondrial ellipsoid improves optical coupling between the inner segment and oil droplet. In contrast, unpigmented droplets found in violet-sensitive cones double sensitivity at its peak relative to other cone types. Oil droplets and ellipsoids both narrow the angular sensitivity of single cone photoreceptors, but not as strongly as those in human cones.

## 1. Introduction

Several adaptations in the eyes of birds suggest that they have well-developed colour vision [1–3]. Their retinae contain four distinct spectral types of single cone with maximum sensitivity to wavelengths in the violet (or ultraviolet), blue, green and red regions of the visible spectrum. In addition, all single cones incorporate an organelle called the oil droplet at the distal end of the inner segment. The oil droplets of blue-, green- and red-sensitive cones contain a mixture of carotenoid pigments unique to each spectral class [2,4]. These oil droplets serve as coloured filters that tune their respective cone spectral sensitivities, thereby improving colour discrimination [3]. Moreover, the spherical shape of oil droplets has led to suggestions that they act as light-collecting lenses that improve photon catch in the outer segment [5–8].

Vertebrate photoreceptors also contain a dense aggregate of mitochondria within the inner segment known as the ellipsoid. The ellipsoid immediately precedes the oil droplet in the intracellular light path, and it has been hypothesized to have an optical effect, including some waveguiding capacity [9,10].

A few previous studies have treated the optics of light collection by oil droplets in bird and turtle cone photoreceptors. Govardovskii *et al*. [5] were the first to model the cone. Illuminating scaled-up physical models with microwave radiation, the authors calculated the influence of the oil droplet and ellipsoid on the transmission of light into the outer segment. They found that both oil droplets and ellipsoids increase the light transmission under axial illumination, but narrow the angular acceptance of the photoreceptor.

Ives *et al.* [6] and Young & Martin [7] used analytical Mie scattering formulae to study isolated oil droplets of the turtle (*Trachemys scripta elegans*) and pigeon (*Columba livia*), respectively. Both studies found that oil droplets increased field intensity in the area occupied by the outer segment. Ives *et al*. also considered the refractive index of the oil droplet as a function of wavelength. However, neither considered the optical effect of the outer segment on the difference in light collection with and without an oil droplet.

Recently, Stavenga & Wilts [8] used the approach of finite-difference time-domain (FDTD) optical modelling, showing that the carotenoid pigments present in oil droplets alter the refractive index as a function of wavelength and hence the focusing properties of the isolated oil droplet.

In this work, we address the collective optics of the inner and outer segment components by modelling the optical performance of the complete single cone photoreceptors using FDTD. To do this, we take into account detailed optical parameters of the oil droplet, outer segment and mitochondrial ellipsoid, all experimentally measured using serial block face scanning electron microscopy (SBFSEM), digital holographic microscopy (DHM) and microspectrophotometry (MSP). We consider refractive and anomalous dispersive effects in the cone oil droplet, as well as reflections at its interface and how these influence cone sensitivity. Furthermore, we use simulations to predict the influence of the oil droplet and ellipsoid on the acceptance angle of the different spectral types of cone photoreceptors.

## 2. Methods

### 2.1. Block face scanning electron microscopy

Two five-week post-hatch chickens (*Gallus gallus domesticus*) were sacrificed in the bright light adapted state by carbon dioxide asphyxiation, approved by the local Swedish ethical board. Eyes were enucleated, and samples (approx. 1 cm^2^) of the central retina were cut out and fixed in 2% glutaraldehyde : 2% paraformaldehyde, 2 mM calcium chloride in 0.15 M cacodylate buffer, following a standard protocol [11]. Samples were transferred to cacodylate buffer with 2 mM calcium chloride and stored for a week. The samples were treated with potassium ferrocyanide and aqueous osmium tetroxide, washed with double distilled water (ddH_2_O) and treated again with aqueous osmium tetroxide. After a second wash in ddH_2_O, the samples were stained with aqueous 1% uranyl acetate and then with lead aspartate. Samples were then dehydrated in an alcohol series and acetone, and embedded in Durcupan resin. Electron microscopy of the retinal samples was performed at the electron microscopy unit at Helsinki University, Finland, using a block face scanning electron microscopy technique, 3-View (Gatan, Pleasanton, CA, USA).

### 2.2. Morphological and refractive index measurement of single cones

Four eight-week post-hatch chickens were sacrificed by carbon dioxide asphyxiation, following 2 h of dark adaptation, under the ethical guidelines of the University of Bristol. The eyes were removed under dim red light and hemisected with a stainless steel razor blade; the vitreous body was carefully removed and the posterior hemisphere immediately placed in phosphate-buffered saline (Sigma-Aldrich, St Louis, MO, USA). Pieces of the retina, approximately 2 mm in diameter, were fixed for 10 min in 4% paraformaldehyde, 5% sucrose solution at 37°C. Photoreceptors were dispersed by repeated pipetting and applied to a glass microscope slide coated with poly-l-lysine (Sigma-Aldrich).

Optical properties of the photoreceptors were measured using a digital holographic microscope (DHM; T1000, LyncéeTec, Lausanne, CH) at a laser wavelength of 660 nm. Holographic data were reconstructed using DHM software, Koala (version 4; LyncéeTec) and phase data processed using Matlab (version 8.3; MathWorks, Natick, MA, USA). To calculate the refractive indices of oil droplets, outer segments and ellipsoids, linear profiles in phase retardation were fitted to simulated phase retardation data covering the relevant refractive index and size range at the DHM laser wavelength. This method was validated against a control sample of 2 µm mean diameter polystyrene microspheres (Polysciences, Warrington, PA, USA) suspended in water and showed no significant difference between the published and measured refractive index (1.585) [12] versus 1.596 ± 0.04, respectively (*t*_19,20_ = 1.192, *p* = 0.248). Organelle dimensions were also measured from the reconstructed phase images in addition to bright-field and fluorescence microscopy images and SBFSEM micrographs.

### 2.3. Oil droplet microspectrophotometry

Absorbance spectra of oil droplets were measured using the ‘expanded oil droplet’ method described previously [4,13]. Frozen retinae from two-week post-hatch chicks were thawed, and the cells lysed by immersion in distilled water and brief vortexing. The homogenate was then centrifuged at 10 000*g* for 2 min, resulting in the accumulation of oil droplets at the water surface. Droplets were collected, placed on concanavalin A (Sigma-Aldrich)-coated quartz coverslips and dried. Once dry, droplets were covered with pure glycerol (Sigma-Aldrich) for further manipulation and measurement. Drying was necessary to affix the droplets to the slide for the expansion procedure. Glycerol was used as a medium to reduce refractive index contrast of the droplets and the media to reduce scatter of the measurement beam. The absorptance spectra of the oil droplets were measured in the wavelength range 350–700 nm using a custom-built microspectrophotometer (MSP) described elsewhere [14]. The diameter of the droplets was measured before and after expansion. Oil droplets were fused with a larger (5–20 µm diameter) droplet of light mineral oil (Sigma-Aldrich) that diluted the dense pigmentation and allowed for detailed resolution of the droplet absorbance spectrum and measurement of peak absorbance. Peak optical density *in vivo* was calculated from the expanded droplet measures, using geometrical considerations and assuming no absorption by the mineral oil [4,13].

### 2.4. Electromagnetic simulations

Electromagnetic simulations were undertaken with the freely available FDTD software MEEP [15], using the computational facilities of the Advanced Computing Research Centre, University of Bristol. FDTD allows numerical calculation of electromagnetic fields in complex geometries under illumination by a specified light source. Field intensity visualizations were processed in the open-source software, Paraview (www.paraview.org). Cuboidal simulation cells were set up with perfectly matched layers (PMLs) of 1 µm thickness on each face removing reflections and simulation edge effects in addition to a 1 µm space between the model and the PML along each cardinal axis (*x*, *y*, *z*). A schematic of the simulation environment is presented in electronic supplementary material, figure S2. Simulations used plane-polarized white light sources with Gaussian time dependence. Angular acceptance calculations were made under illumination from a plane wave source rotated about the closest edge of the structure with a constant straight-line distance perpendicularly from the source to the structure. The surface area of the source was kept constant for all calculations. The cone photoreceptor was modelled as a cylinder (representing the ellipsoid), absorbing sphere (oil droplet) and second cylinder (outer segment) based on the morphological measurements of this study. Dimensions used were mean values measured from eight-week post-hatch chickens as described above. The refractive index of the medium surrounding the photoreceptor was 1.35, according to previously calculated values for intra-photoreceptor media [16]. All materials were modelled as optically linear, isotropic media, and only the oil droplet was modelled as absorbing, because weak absorption by the visual pigment has been shown to have no significant effect on the refractive index [17,18]. Calculations were made at a resolution of 34 computational cells per µm distance, above which solutions converged.

Oil droplet absorption coefficients were calculated from MSP data according to2.1
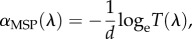
where *α*_MSP_(*λ*) is the absorption coefficient in µm^−1^ as a function of wavelength, *λ*; *d* is the mean droplet diameter in µm and *T*(*λ*) is the mean measured transmittance. The Beer–Lambert law is assumed to hold. In MEEP, the complex dielectric function *ɛ* is modelled using a sum of a series of Lorentzian dipole oscillators. The dielectric function determines the refractive and absorptive properties of the dielectric medium. To model *ε*, oil droplets were given dielectric functions of the form2.2
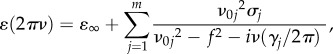
where *ɛ*_∞_ is the frequency-independent component of *ɛ*; *ν* represents frequency; *ν*_0*j*_ is the *j*th dipole central frequency; *m* is the number of Lorentzian dipoles; *γ_j_* is oscillator strength of the *j*th dipole and *σ_j_* is a scaling parameter (see electronic supplementary material, figure S3) [15]. The refractive index, *n*, extinction coefficient, *κ*, and modelled absorption coefficient, *α*_model_, were determined from *ɛ* using the method described in the electronic supplementary material using the relations2.3
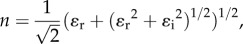
2.4
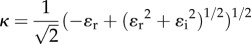
2.5
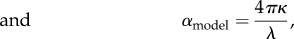
where *ɛ*_r_ and *ɛ*_i_ are the real and imaginary parts of the dielectric function, respectively [19]. Parameters of the dipole model of the dielectric function (*ν*_0*j,*_ *γ_j_* and *σ_j_*) were determined by fitting *α*_model_ to *α*_MSP_, such that the model function of *n* equalled the mean of measured refractive indices in each oil droplet class at the measurement wavelength, 660 nm. The effects of normal dispersion on the wavelength-dependence of the refractive index were ignored and assumed to be negligible in comparison with the effect of anomalous dispersion. All structural elements were treated as rotationally symmetric isotropic dielectrics.

Light transmission was quantified from FDTD simulations by calculating the relative transmission of light through a plane covering the outer segment cross section placed 1 µm from the oil droplet in the light propagation direction. Relative flux, *Φ*(*λ*), was calculated by2.6
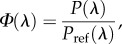
where *P*(*λ*) is the Poynting flux [15] in the presence of optical structures (i.e. outer segment, oil droplet and ellipsoid) and *P*_ref_(*λ*) is the reference Poynting flux calculated when no optical structures are present. The relative sensitivity of cones was calculated by two distinct methods as a comparison of calculating sensitivity with and without optical considerations. Cone relative sensitivities, *S*_1_(*λ*) and *S*_2_(*λ*), were calculated by2.7
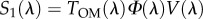
and2.8
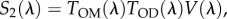
where *T*_OM_ and *T*_OD_ are the ocular media [20] and oil droplet transmittances, respectively. *V*(*λ*) is the visual pigment template for A1-chromophores [21] using maximal absorbance wavelength (*λ*_max_) values published previously [22]. *V* differs for each cone type, because each expresses a different visual pigment.

To gain an indication of the contribution of oil droplet reflectance to the modulation of cone sensitivity, the reflectance of the front hemisphere surface of the oil droplet was calculated using the Fresnel equations for reflectance (see the electronic supplementary material).

Angular sensitivity, *Λ*(*θ*) as a function of light propagation angle, *θ*, was calculated by2.9
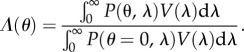
To calculate the half-width of angular sensitivity, Gaussian curves were fitted to angular acceptance data by linear regression and the angle of half-maximum transmission found.

## 3. Results

### 3.1. Morphology and optical properties of single cones

**Figure 1.**
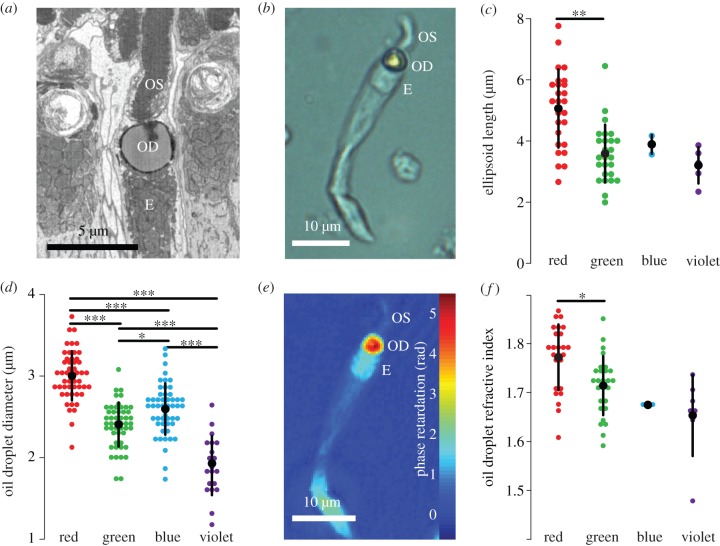
Morphology and refractive indices of the chicken single cone photoreceptor. (*a*) A single cone from serial block face scanning electron microscopy shows the E, ellipsoid; OD, oil droplet and OS, outer segment. (*b*) A dissociated green single cone under bright-field microscopy. (*c*) Lengths of the ellipsoids for all four cone types measured by bright-field microscopy. (*d*) Oil droplet diameter for all four cone types measured by bright-field microscopy. (*e*) A phase retardation image of a dissociated single green cone (seen in bright field in (*b*)). (*f*) The real part of the oil droplet refractive index measured at 660 nm. Only the difference between the red and green cone is significant (*p* = 0.011). Large black dots show mean values; error bars represent 1 s.d. Significance bars show the results of Tukey's honest significant difference (HSD) test with the following *p*-values: *p < 0.05, **p < 0.01, ***p < 0.001.

In order to build representative models of the single cones of the chicken, morphological dimensions and refractive indices of oil droplets, ellipsoids and outer segments were directly measured along with the absorbance spectra of the oil droplets. Using block face scanning electron microscopy and bright-field light microscopy of dissociated photoreceptors (figure 1*a,b*), several morphological properties were found to be conserved across the four spectral types of cone. Oil droplets were seen to be spherical and the outer segments cylindrical for the portion closest to the oil droplet. The mitochondrial ellipsoids surrounded the entire front hemisphere of the oil droplet. They had a flat interface at their inner end (figure 1*a*) and an equal cross-section to the oil droplet (figure 1*a,b*). However, not all properties were uniform across the cone types. The length of the ellipsoids and diameter of the oil droplets (figure 1*c,d*) in the red cone were found to be significantly larger than those in other cone types. Outer segment diameters did not vary significantly between cone classes with a mean diameter of 1.73 ± 0.56 µm (ANOVA, *F*_3,43_ = 0.705, *p* = 0.597).

Next, the real part of the refractive indices of the oil droplets in all spectral types of cone was quantified from the phase imaging data obtained by DHM at 660 nm (figure 1*e,f*). The refractive indices were different between each cone type, with mean oil droplet refractive index following the relationship: *n*_red_ > *n*_green_ > *n*_blue_ > *n*_violet_ (figure 1*f*). The oil droplet refractive index of the red cone was significantly higher than in the green cone (ANOVA, *F*_3,62_ = 8.015, *p* < 0.001; Tukey's honest significant difference (HSD), *p* = 0.011). Although small sample sizes precluded statistical comparison in the blue and violet cones, the trend was maintained across all cone types. We also found that within the inner segment, only the ellipsoid had an elevated refractive index (figure 1*e*). The refractive index of the ellipsoid, 1.43 ± 0.05 (ANOVA, *F*_3,54_ = 0.536, *p* = 0.660), and outer segment, 1.45 ± 0.05 (ANOVA, *F*_3,43_ = 0.459, *p* = 0.713), did not vary significantly between cone types.

**Figure 2.**
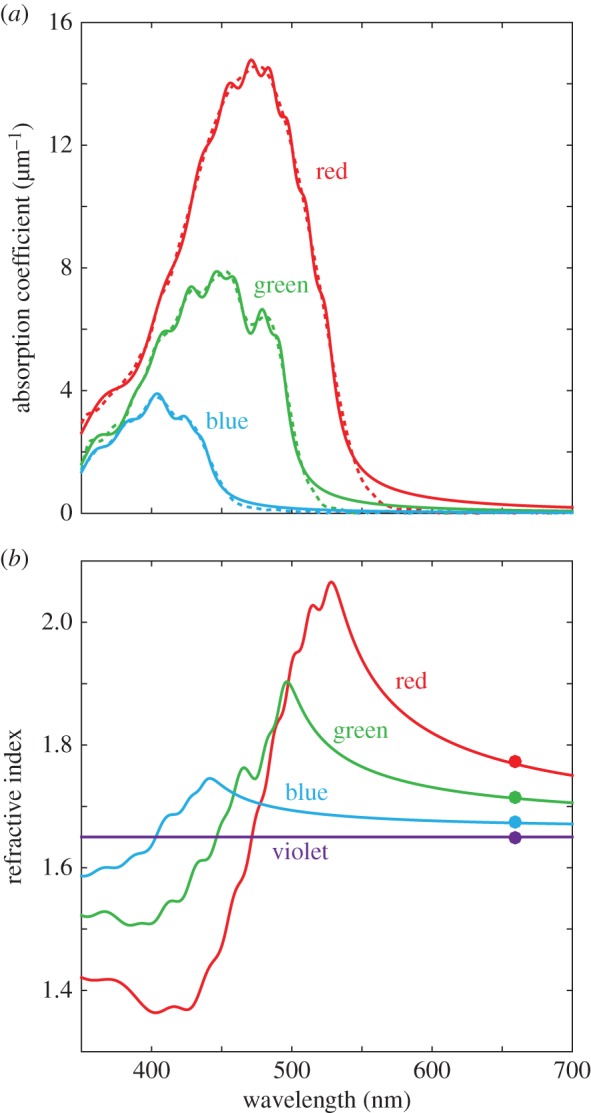
Optical characterization of the oil droplets of single cone photoreceptors. (*a*) Absorption coefficients of the four single cone types: dashed lines are the measured absorption coefficients; solid lines are the simulated absorption coefficients based on Lorentzian dipoles. (*b*) Refractive index of single cone oil droplets: solid lines are the simulated dispersion curves based on Lorentzian dipole absorbers; filled circles are the mean values from DHM measurements. Red cone, red; green cone, green; blue cone, blue; violet cone, violet.

Absorbance spectra, used in modelling the oil droplet refractive index as a function of wavelength, were measured by MSP using the expanded oil droplet method [4,13]. The spectra showed a progressively longer cut-off wavelength correlated with the peak absorption of the visual pigment of the corresponding cone type (figure 2*a*), as previously described [2,4,23–26]. The spectra of the pigmented oil droplet types were consistent with the presence of one of three major carotenoid pigments: astaxanthin (red cone oil droplets), zeaxanthin (green) and galloxanthin (blue); however, all oil droplets showed some degree of mixing of carotenoid types. There was also no measurable absorption across the wavelength range 350–700 nm in the violet cone oil droplet, as has been previously reported [2,4,23–26]. Absorption coefficients, refractive indices measured by DHM at 660 nm and equations (2.1)–(2.5) were used to calculate the refractive index of the oil droplet as a function of wavelength (figure 2*b*). Deviations from the frequency-independent value of the refractive index owing to absorption were higher in oil droplets with the highest absorption coefficients (figure 2*b* and electronic supplementary material, figure S4).

### 3.2. Cone light transmission

**Figure 3.**
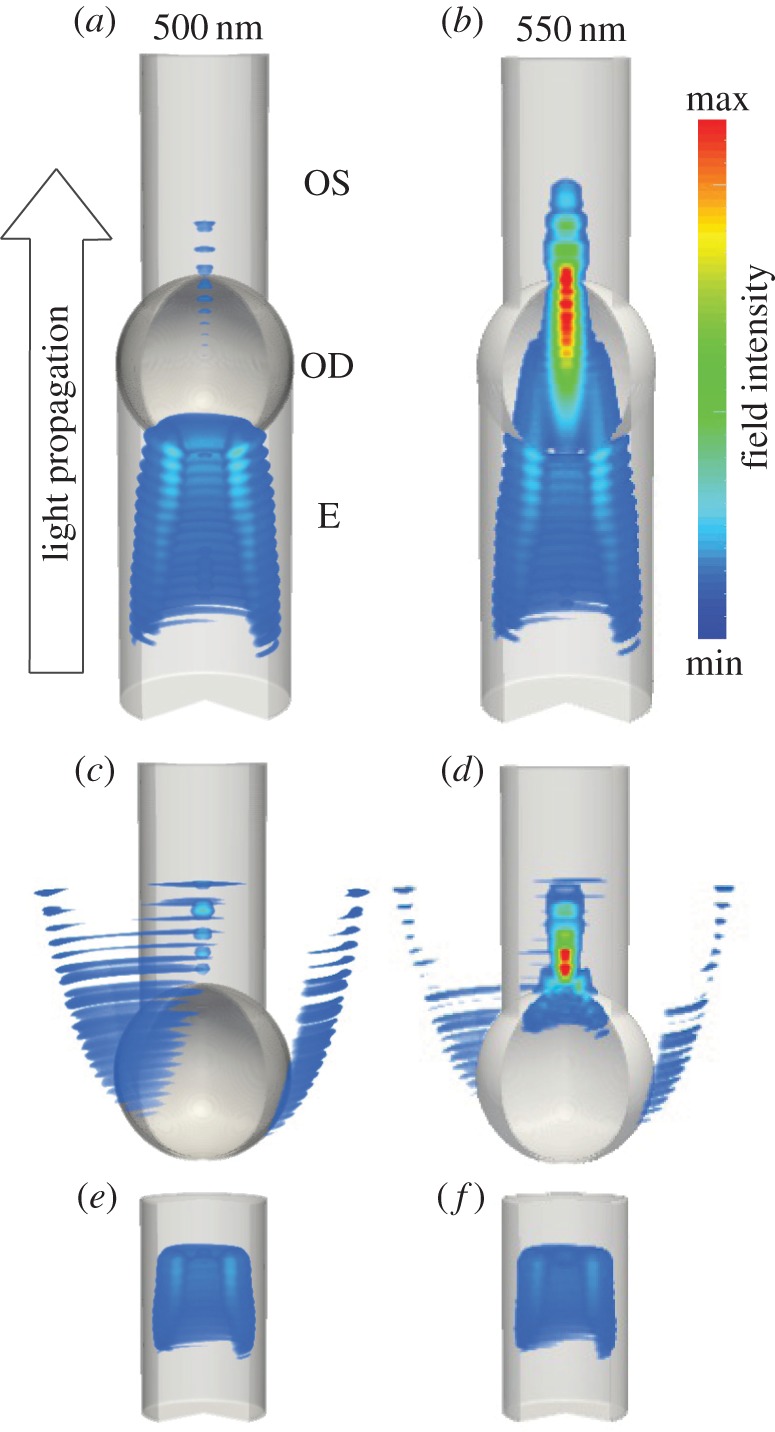
Simulated field intensity under monochromatic plane illumination within the ellipsoid (E), oil droplet (OD) and outer segment (OS) of the green single cone photoreceptor. The wavelength of illumination is 500 nm (*a*, *c* and *e*) 550 nm (*b*, *d* and *f*). Structures represent: full receptor model (*a*,*b*), no ellipsoid (*c,d*) and outer segment only (*e*,*f*).

Using measured morphological and refractive index data, we sought to understand the optical influence of the cone photoreceptor inner and outer segment structure on the spectrum of light transmitted into the outer segment. Accordingly, we used the refractive indices, spectral absorbance of the oil droplets and morphological measurements to build a series of FDTD simulations to determine relative light flux into the outer segments. Calculations were made for the three model structures shown in figure 3: outer segment alone; outer segment and oil droplet; the outer segment, oil droplet and ellipsoid together. Visualizations of the spatial distribution of field intensity were also used to provide insights into the mechanisms underlying differences in transmission (figure 3).

**Figure 4.**
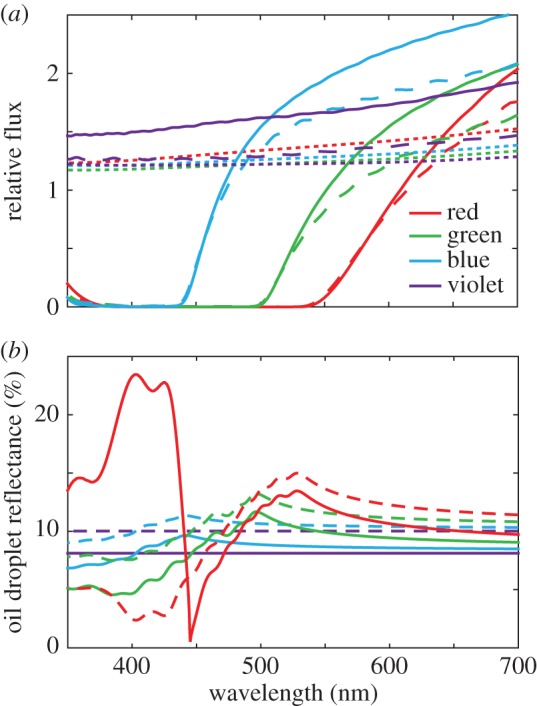
Optical transmission in single cone photoreceptors. (*a*) Relative flux calculated in FDTD simulations for differing combinations of organelles; dotted lines show outer segment only, dashed lines with an oil droplet and solid lines are with all three elements. (*b*) Percentage reflectance of the front hemisphere of the oil droplet of each cone type. Dashed lines show reflectance without an ellipsoid (*n*_1_ = 1.35) and solid lines with an ellipsoid (*n*_1_ = 1.43). The discontinuity in the curve for the red cone oil droplet in the presence of the ellipsoid (solid red line) below 450 nm occurs where *n*_1_ > *n*_2_.

For the outer segment alone, there was a more than 20% increase in transmission owing to waveguiding in the first 1 µm of the outer segment (figure 4*a*, dotted lines). With the introduction of the oil droplet, the relative flux into the outer segments dropped steeply in the spectral regions of oil droplet absorption in the blue, green and red cone models (figure 4*a*, dashed lines). Interestingly, we found that the flux into the outer segments is also reduced at wavelengths longer than those absorbed by the oil droplets. The addition of the ellipsoid (figure 4*a*, solid lines) reduced some of these losses owing to the improved refractive index matching across the ellipsoid–oil droplet boundary, and this effect is seen most clearly in the violet cone model where the impedance matching improves the coupling into the outer segment by approximately 16% across the spectral sensitivity range.

Calculations were also performed to assess the reflectance of the oil droplet interface (figure 4*b*). Reflectance was lower in all cases with the presence of the ellipsoid owing to a reduction in refractive index contrast. The highest reflectance was seen where the refractive index was highest, reaching 13% at 525 nm in the red cone. A very high reflectance is seen for wavelengths less than 450 nm in the red cone. This occurs where the oil droplet refractive index is less than that of the ellipsoid, meaning that at high angles of incidence (at the edge of the oil droplet), total internal reflection phenomena have a large contribution to reflectance. Reflections reduce light transmission between the inner and outer segment. For instance, at 600 nm where the absorption coefficient of the oil droplet of the red cone is less than 1 µm^−1^, its reflectance is more than 10%.

**Figure 5.**
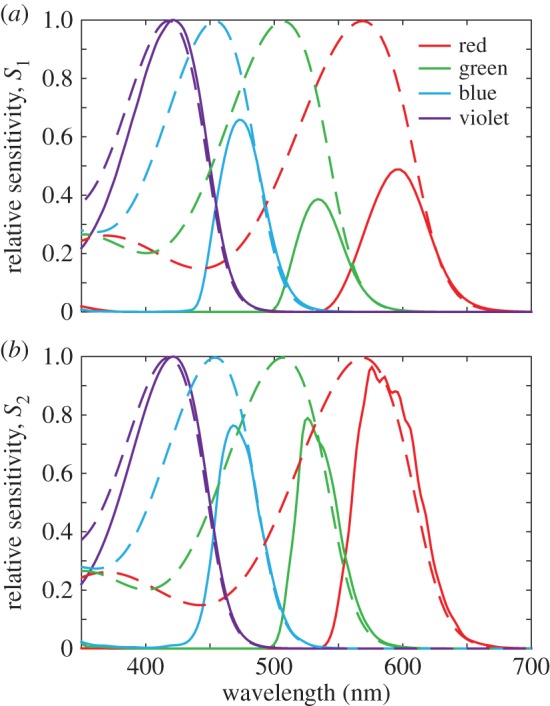
Relative sensitivities of the single cone photoreceptors calculated using two contrasting methods. (*a*) Relative sensitivities (solid lines) accounting for optical effects as shown in figure 4*a*, visual pigment absorbance template (dashed lines) and ocular media transmittance (equation (2.7)). (*b*) Relative sensitivities (solid lines) calculated by conventional methods using oil droplet transmittance, ocular media transmittance and visual pigment absorbance template (dashed lines) (equation (2.8)). Relative sensitivity curves are normalized to the maximum in the violet cone in each case.

Next, cone sensitivities for the complete cell model were calculated using relative flux spectra, visual pigment templates [21] and the ocular media transmission spectra (equation (2.7); figure 5*a*) [20]. The green and red cones were predicted to have similar relative sensitivities at their peak, whereas the blue and violet cones had predicted relative sensitivities of approximately 1.5 and two times those of the green and red cones, respectively (figure 5*a*). Figure 5*b* shows spectral sensitivity data predicted by methods from prior studies [3,20,25,27,28] where only the oil droplet transmittance measured by MSP, ocular media transmittance and the visual pigment template are used to calculate the visual sensitivity (equation (2.8)). While the predicted relative sensitivities for blue, green and red cone models were similar between our results and the previous models (figure 5*b*), the relative sensitivity of the violet cone was substantially higher in simulated sensitivities (equation (2.7); figure 5*a*) than in models calculated according to equation (2.8) (figure 5*b*).

### 3.3. Determination of acceptance angles of single cone photoreceptors

**Figure 6.**
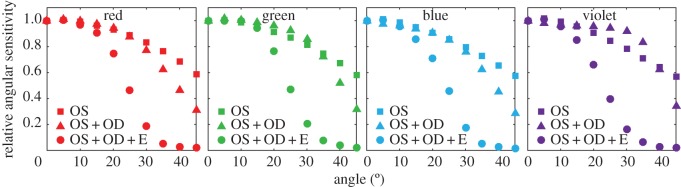
Angular sensitivity of single cone photoreceptors. Relative transmission of light into the outer segment for plane wave illumination from an angle, calculated relative to on-axis case integrating across the visible spectrum. Data are shown for structures including the outer segment (OS), oil droplet (OD) and ellipsoid (E). A sharp reduction in angular sensitivity is seen on the addition of the ellipsoid.

Light arrives at the retina from a wide angular spread, determined by the focal properties of the cornea and lens, as well as the pupil diameter. FDTD calculations were again undertaken for the same three different models as above: outer segment alone, outer segment and oil droplet; outer segment, oil droplet and ellipsoid together, in order to determine the effect the intracellular elements have on the acceptance angle of the single cones. In all three sets of calculations, flux into the outer segment was seen to decrease as expected according to a roughly Gaussian dependence as a function of an increasing propagation angle relative to the photoreceptor transmission axis (figure 6). Moreover, similar patterns were observed in simulations across all four cone types. However, the ellipsoid had a significant effect on the acceptance angles of all the photoreceptors with its addition providing a reduction of the half-maximum angle to near approximately 22° (LWS, 22.6°; MWS, 23.2°; SWS, 22.2°; VS, 21.2°).

## 4. Discussion

We have constructed a detailed optical model of the four avian single cone types to calculate the light transmittance from the inner segment to the outer segment. This has allowed us to test long-standing predictions concerning the optical effect of the oil droplet and mitochondrial ellipsoid. To the best of our knowledge, this represents the most complete optical modelling of vertebrate photoreceptors to date.

### 4.1. Morphology and optical properties of single cone photoreceptors

Phase imaging by DHM showed that the mitochondrial ellipsoid, oil droplet and outer segment are the principal refractive elements of the single cone photoreceptor (figure 1*e*). We also observed by SBFSEM that the ellipsoid has approximately the same diameter as the oil droplet (electronic supplementary material, figure S1), suggesting that both structures together set the aperture diameter of the photoreceptor. Consistent with previous observations of avian single cones, the outer segment was seen to be cylindrical and not tapered [29]. In addition, the size distribution of oil droplets was similar to what has been previously reported in birds [4,27].

Refractive indices of cone oil droplets displayed a similar pattern to prior observations in the red-eared slider (*T. scripta elegans*) [6] of reducing across the classes (figure 1*f*) and were higher than the value of 1.48 reported in the pigeon (*C. livia*) [7]. However, the values measured here take the range 1.65–1.77, substantially higher than the range reported in the turtle of 1.48–1.69 [6]. We expect these values to be accurate, owing to good agreement between our measurements of the polystyrene control sample and previous measurements of the same material at the same wavelength reported above [12].

### 4.2. Oil droplets and ellipsoids influence light transmission

The previous literature had suggested that all oil droplets act as light-collecting lenses to increase the delivery of on-axis propagating light into the outer segment, by up to six times in some cases [5–8]. Our modelling suggests that in chickens, only the transparent oil droplet in the violet cone fits with this conclusion, increasing the transmission of light for wavelengths to which the violet cone is sensitive, by a factor of 1.5–2 (figure 4*a*). In contrast, the pigmented oil droplets reduce light delivery to the outer segment by as much as 20–30% at wavelengths to which the cone is sensitive (figure 4*a*).

Clearly, there appears a difference here between our results and the literature. However, the difference is due to two main reasons: (i) the reflection of light from the oil droplet–inner segment interface, caused by the abrupt transition in the refractive index that occurs at the boundary of the carotenoid-pigmented droplets (figure 4*b*), is greater in the chickens owing to the higher refractive indices of the oil droplets compared with turtles [6]. (ii) Moreover, the calculations of the increase in transmission in turtles [6] used oil droplets of around 6–14 µm in diameter and outer segments of 1.5–2.5 µm in diameter at their start [6,13,30]. In the chicken, the oil droplets are only 2.5–3.4 µm in diameter and the outer segments approximately 1.73 µm as measured in this study. Such a smaller diameter, and therefore, a smaller effective collection area would reduce the relative transmission. We should stress here the importance of interspecific variation. In some birds, such as the wedge-tailed shearwater (*Puffinus pacificus*), the oil droplets of the central retina have much lower pigment densities, and therefore may have lower refractive indices, than those in the peripheral retina [25]. This reduced refractive index may improve coupling into narrow central retina photoreceptors by reducing the reflectance of the ellipsoid–oil droplet interface. A central-peripheral gradient in oil droplet pigmentation density has also been observed in the sacred kingfisher (*Todiramphus sanctus*) [31]. Furthermore, dorsoventral gradients in oil droplet pigmentation density are common among birds (albeit most strongly within double cone oil droplets) [26,32], which undoubtedly affects light coupling between the inner and outer segment.

The ellipsoid has previously been shown in species without an oil droplet to have an elevated refractive index and has also been theorized to improve light catch via a lensing effect [9,10,33]. In the cones of chickens, the ellipsoid is approximately the same diameter as the oil droplet (*t*_64.8,38_ = 1.46, *p* = 0.150, see electronic supplementary material, figure S1); thus, it cannot capture light from a wider area. However, the high refractive index of the ellipsoid does reduce some of the light loss at the oil droplet via a reduced contrast in the refractive index at the oil droplet boundary. The presence of the ellipsoid improves transmittance by around 25% in all cone types (figure 4*a*). In the case of the unpigmented oil droplet in the violet cone, our results do show similarities to the microwave models used by Govardovskii *et al*. [5] which also showed that an unpigmented oil droplet and ellipsoid increase the transmission of light into the outer segment for on-axis propagating light.

### 4.3. Contributions of optical structures to single cone sensitivity

The capacity to predict the transmittance into the outer segment based on whole cell morphology allowed a further investigation into the spectral sensitivity of the bird colour vision system. The principal consequence of the gain or loss of the light transmitted into photoreceptor outer segments depending on the optical properties of the unpigmented and pigmented oil droplets, respectively, is the creation of a mechanism for controlling the relative sensitivities of those cone classes. In our results (figure 5*a*), the violet cone type is optically predicted to be more than twice as sensitive at its peak than the other three cone types where this enhanced sensitivity is due to relatively greater transmittance caused by the collection of light into the outer segment by the transparent oil droplet. The different levels of transmission in the other cone types that contain the pigmented oil droplets set the relative levels of sensitivity in each colour channel. Traditionally, the method of predicting relative cone sensitivities involves multiplying the oil droplet and ocular media transmittance by the visual pigment absorbance (figure 5*b*) [3,20,25,27,28]. However, we find here that the optics of the complete photoreceptors, including the optics of ellipsoid and the oil droplet, has a considerable influence on the relative spectral sensitivities of the single cones (figure 5*a*).

Measures of spectral sensitivity in the chicken have been made via electroretinography [34,35] and behavioural tests [36]. These tests have found highest sensitivity to be in the medium-to-long wavelength regions of the spectrum. This contrasts with calculations of individual cone sensitivity shown here (figure 5*a*), in which the highest sensitivities are in the violet and blue cones. The difference here is that we consider the sensitivity of a single cone, rather than the complete eye, as considered in other studies [34–36]. The explanation of the difference may be found in the relative abundances of the cone types or background adaptation conditions in behavioural experiments. Red and green cones are the most abundant single cone types at approximately 29% and 35% of single cones, respectively, in comparison with the blue and violet cones which only represent 21% and 15%, respectively [37]. The relative abundance of cone types may even highlight the reason for the necessity for improved sensitivity in the violet and blue cones, because fewer are available in the retina to take advantage of spatial pooling to improve the signal-to-noise ratio in these spectral channels.

Improvements in transmission in the violet cone may be beneficial, because even in daylight, violet and ultraviolet wavelengths are comparatively scarce in certain environments, such as the forest [38,39].

### 4.4. Angular acceptance of single cones

The ellipsoid and oil droplet also impact the directional light collection properties of the cone photoreceptor. The oil droplet and ellipsoid together were found to reduce the angular sensitivity of the cone to a half-maximum angle between 21° and 25°, narrowing the directionality of the outer segment from a half-maximum angle of more than 30°.

Angular acceptance in dielectric waveguides is governed by the contrast in the refractive index between the waveguide and the surrounding medium, as well as the diameter of the waveguide (for the case of single-mode waveguides) [40,41]. Although the addition of the ellipsoid narrows the angular sensitivity relative to a model without one, for either a larger difference between the interior and surrounding refractive index, or for a narrower waveguide, the angular acceptance angle increases. Thus, the higher refractive index of 1.43 in the ellipsoid compared with 1.353 in human cones may explain the higher acceptance angle of these photoreceptors relative to previous studies [40,42]. Furthermore, because the eyes of chickens have relatively shorter focal lengths, larger pupils and lenses than humans, wider acceptance angles are better matched to receive light reaching the retina from a wider angular range [43].

## 5. Conclusion

In summary, we have studied optical transmission in the single cone photoreceptors of the chicken. The data clearly indicate that it is important to consider the optical properties of photoreceptors as a whole, both the intracellular structures in the inner segment, the ellipsoid and the oil droplet, and the outer segment. These elements shape the spectral variation in light transmission, not only through absorption, but via differences in the efficiency of light coupling borne from variation in the refractive index and morphology. Owing to the strong absorption of pigments in the oil droplets, the refractive index is affected outside the spectral regions where absorption occurs and the observed effect is that pigmented oil droplets reduce the flux of light into the outer segment. The non-absorbing oil droplet of the violet cone, having a lower refractive index, is capable of increasing flux. The ellipsoid, having a refractive index between that of the cytosol and the oil droplet, improves the transmission of light by reducing refractive index contrast. Furthermore, both the oil droplet and ellipsoid reduce the angular sensitivity of the cone photoreceptor. Overall, we find that the optical properties of the inner segment organelles have a controlling influence on light transmission in each spectral class of cone and hence their relative sensitivities.

## Supplementary Material

Electronic supplementary material

## Supplementary Material

Raw simulation data

## Supplementary Material

Oil droplet morphological and refractive index measurements

## Data accessibility

The datasets supporting this article have been uploaded as part of the electronic supplementary material.
