# Supplementary material for: Optics of cone photoreceptors in the chicken (Gallus gallus domesticus)
Source: J R Soc Interface. 2015 Oct 6;12(111):20150591. doi: 10.1098/rsif.2015.0591 (PMC4614498; doi:10.1098/rsif.2015.0591)
Supplement: Electronic supplementary material [file rsif20150591supp1.doc]

**Electronic supplementary material**

The electronic supplementary material for this paper comprises this document and a spreadsheet of raw data. This document includes measurements of the oil droplet and ellipsoid diameters; a schematic of the simulation geometry; calculations of the oil droplet dielectric function using both the Lorentzian model; and the Kramers-Kronig relations and calculations of the oil droplet reflectance.

The raw data spreadsheet includes measurements of the morphology and phase retardation of the oil droplets, ellipsoids and outer segments. Phase measurements are characterized by their full-width at half-maximum (‘FWHM’ in the spreadsheet) and peak height (‘Peak Phase’).

**Oil droplet and ellipsoid diameters**

Oil droplets and ellipsoids were measured in SBFSEM images to have very similar diameters (t_64.8,38_=1.46, p=0.150).

**Figure S1**

Diameters of oil droplets and ellipsoids of single cones measured from SBFSEM images of tissue from the central retina of the chicken. Black dots show individual paired measurements; black line represents the result of a linear regression with slope of 0.8463 and intercept of 0.5559. Sample size is 38.

**Simulation Geometry**

**Figure S2**

Schematic of single cone simulation environment. Central cross-section of the 3-dimensional electromagnetic simulation cell.

**Lorentzian Dipole Model of the Dielectric Function**

Absorption and dispersion in the FDTD software MEEP is represented using a sum of Lorentzian dipoles, which determine the dielectric function, ε , and hence the real part of the refractive index, *n* and the extinction coefficient, κ which govern the refractive and absorptive properties of optical media. The dielectric functions of oil droplets were modeled using this method and the real and imaginary parts of ε for the oil droplets of the red, green and blue-sensitive cone models are shown in figure S3.

**Figure S3**

Lorentzian oscillators used to model oil droplet dielectric function. The real (a,b, & c) and imaginary (d, e & f) parts of the dielectric function in the oil droplets of the LWS (a&d), MWS (b&e) & SWS (c&f) cones. Narrow solid lines show the individual Lorentzian contributions (ε_m_), dashed lines show the frequency independent component (ε_∞_) and broad solid lines show the addition of all these components (ε).

**Kramers-Kronig Relations**

The relationship between the real part of the complex refractive index, *n* and the imaginary part κ is governed by the Kramers-Kronig relations. If either *n* or κ is known, the other may be calculated. The Kramers-Kronig relations were used as a comparison to the method of calculating *n* and κ used in the main text. The contribution to *n* due to absorption, *Δn* is given for the frequency domain by Ohta & Ishida (1988) as

|  | 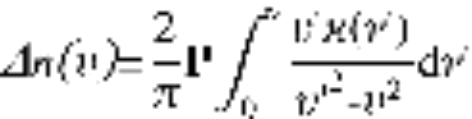 | (S1) |
| --- | --- | --- |

where *ν* is the frequency, which is related to wavelength, *λ*, by *ν=c/λ* where *c* is the speed of light in a vacuum. **P** denotes that the Cauchy principal value of the integral should be taken, since the integrand goes through a pole at *ν’=ν*. Ohta & Ishida (1988) determined Maclaurin’s formula to be a computationally fast and accurate method for calculating *Δn* for real, discrete data points. In this method, the pole is avoided by only summing every other value in the integrand. A description of the method can be found in Ohta & Ishida (1988). A comparison of the values of *Δn* is shown in figure S4c and agrees well with the method used in the main text.

**Figure S4**

Comparison between Lorentzian and Kramers-Kronig models of the dielectric function. a) Measured oil droplet absorption coefficient (α). b) Extinction coefficient (κ) calculated from the measured absorption coefficient (solid lines) and modelled using the fitted Lorentzian dipole model (dashed lines). c) Deviation in refractive index from the frequency-independent component (∆n) from the Lorentzian dipole model (dashed lines) and calculated from the extinction coefficient using the Kramers-Kronig (K-K) relations.

**Determining refractive index using absorption spectra**

Spectral dependence of the refractive index of oil droplets was determined using the relations shown in the main text in equations 1-5 and following the steps:

1. MSP was used to measure absorption coefficient spectra of expanded oil droplets according to Goldsmith *et al*. (1984).
2. A least-squares regression was used to determine the dielectric function of the oil droplet. Free parameters in equation 2 were *ε*_∞_, *ν*_0_*_j_* , *m* *γ_j_* and *σ_j_*. The result of equation 2 was used to calculate *n* and *κ* using equations 3 & 4. Equation 5 allowed the calculation of a model absorption spectrum, which was fit to the measured spectrum from MSP.
3. The refractive index, *n*, was fit by eye to the mean refractive index at 660 nm measured from DHM (results in main text, fig. 1c).
4. The result of fits of absorption coefficient and refractive index spectra are shown in figure 2.

**Oil Droplet Reflectance**

The reflectance of the first interface of the oil droplet was estimated using the Fresnel equations for reflectance, *R*(*λ*,*θ*) for *s*- and *p*-polarizations as a function of wavelength, *λ* and angle of incidence relative to the surface normal, *θ*

|  | 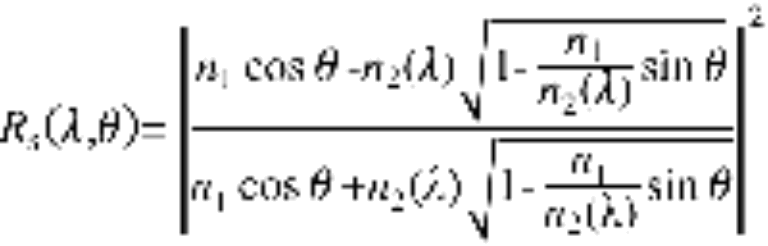 | (S2) |
| --- | --- | --- |

and

|  | 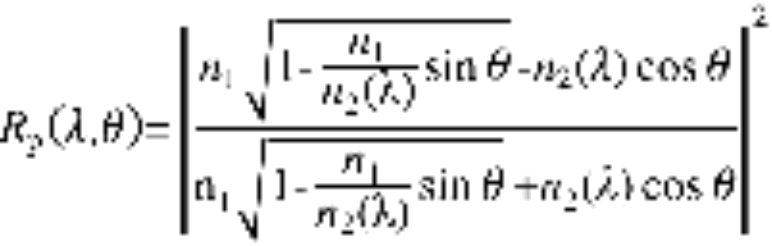 | (S3) |
| --- | --- | --- |

giving the total oil droplet reflectance as

|  | 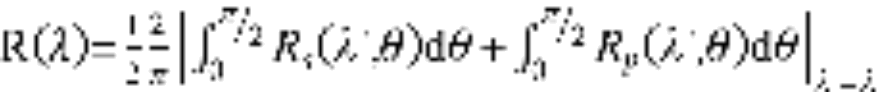. | (S4) |
| --- | --- | --- |

The integrals are evaluated over the range *θ*=0 for the case of a ‘ray’ striking the centre of the oil droplet from an on-axis direction of propagation, i.e. the propagation vector is parallel to the surface normal; and *θ*=π/2 for the case of a ray striking the edge of the oil droplet, at a right-angle to the surface normal. The reflectances for the two orthogonal polarization states *R_s_* and *R_p_* are averaged by addition and multiplying by ½. The function is normalized between the range 0 & 1 (the range of values taken by reflectance) by dividing the total expression by the maximum value of the mean of the integrals, π/2.

**Supplementary References**

Goldsmith, T.H., Collins, J.S., Licht, S. 1984 The cone oil droplets of avian retinas. *Vision Res.* **24**, 1661-1671. (DOI 10.1016/0042-6989(84)90324-9.)

Ohta, K., Ishida, H. 1988 Comparison Among Several Numerical Integration Methods for Kramers-Kronig Transformation. *Appl. Spectrosc.* **42**(6) 952-957.
