# Supplementary material for: Optics of cone photoreceptors in the chicken (Gallus gallus domesticus)
Source: J R Soc Interface. 2015 Oct 6;12(111):20150591. doi: 10.1098/rsif.2015.0591 (PMC4614498; doi:10.1098/rsif.2015.0591)
Supplement: Oil droplet morphological and refractive index measurements [file rsif20150591supp3.gz › Wilbyetal_data_fluxspectra/Notes.rtf]

——————————————————————————————————————————————————————————————Notes on flux spectra data-This zip file contains four folders, one for each cone type. These are the data for both on-axis and angular acceptance calculations presented in figs. 4 & 6 of the paper respectively.-Data files (output from MEEP) follow the naming convention: ‘angle_’ structure ‘-theta-‘ angle of incidence in degrees ‘-‘ structure ‘.dat’ where structure corresponds to the particular simulation.-Data files are tab delimited and consist of four columns.-The first column can be ignored and only contains the prefix ‘flux1:’.-The second column contains the unitless frequency values used by MEEP. To get the wavelength in micrometres, divide 1 by these values.-The third column contains corresponding values of Poynting flux calculated at a plane 1 micrometre into the outer segment, these are the values used to calculate the results in the paper.-The fourth column contains reflected flux values. These are not needed here.——————————————————————————————————————————————————————————————
